# Missed Nursing Care in Neonatal Intensive Care Units for Infants Experiencing or at Risk of Experiencing Substance Withdrawal

**DOI:** 10.1002/nur.70096

**Published:** 2026-07-03

**Authors:** Sooyoung Kim, Adam C. Carle, Rita Pickler, Jin Jun, Heather L. Tubbs‐Cooley

**Affiliations:** ^1^ College of Nursing The Ohio State University Columbus Ohio USA; ^2^ James M. Anderson Center for Health Systems Excellence Cincinnati Children's Hospital Medical Center Cincinnati Ohio USA; ^3^ Department of Pediatrics, College of Medicine University of Cincinnati Cincinnati Ohio USA; ^4^ Department of Psychology, College of Arts and Sciences University of Cincinnati Cincinnati Ohio USA

**Keywords:** missed nursing care, neonatal abstinence syndrome, neonatal intensive care units, nurse workload, substance withdrawal syndrome

## Abstract

Caring for infants experiencing or at risk of substance withdrawal often increases nursing workload and may contribute to missed nursing care (MNC). This study examined the relationship between infants' substance withdrawal status and the occurrence of MNC in neonatal intensive care units (NICUs). We conducted a secondary analysis of data from a longitudinal observational study involving 249 nurses and 1477 infants across 10 U. S. NICUs between April 2021 and May 2023. Nurses completed shift‐level surveys reporting infant conditions and MNC for assigned infants, including whether infants were actively experiencing or at risk of substance withdrawal. Missed nursing care was measured using a 16‐item instrument, and a global indicator captured whether any care was missed. Nurse characteristics were collected at enrollment using a researcher‐developed questionnaire, and infant demographic and clinical characteristics were obtained from the electronic health record. Cross‐classified multilevel logistic regression models were used to account for the clustering of nurses and infants. Compared with infants not identified as at risk of or experiencing withdrawal, infants identified as at risk of or experiencing withdrawal had significantly higher odds of MNC in eight areas, including developmental care, hourly surveillance, airway tube position assessment, high‐risk medication assessment, oxygen titration per protocol, communication during handoffs, pain assessment, and overall missed nursing care. These findings suggest that infants experiencing or at risk of substance withdrawal are particularly vulnerable to MNC, underscoring the importance of accounting for withdrawal risk in nursing workload and staffing decisions to support high‐quality care in NICUs.

## 1 Introduction

In the United States, approximately one infant is diagnosed with opioid withdrawal syndrome every 24 min, equivalent to six infants for every 1000 newborn hospital stays (Centers for Disease Control and Prevention 2024). Substance withdrawal syndrome in infants encompasses a range of problems that occur when a baby withdraws from exposure to narcotic and nonnarcotic drugs in utero or after birth (Hudak et al. 2012). Nationally, the incidence of infants born with substance withdrawal syndrome increased by 82% between 2010 and 2017 (Hirai et al. 2021). Most cases are associated with maternal use of illicit drugs, binge, or heavy drinking during pregnancy (Hudak et al. 2012). Substance withdrawal in infants may result from prenatal exposure, commonly diagnosed as neonatal abstinence syndrome (NAS), or from postnatal administration of opioids or benzodiazepines for analgesia or sedation (Hudak et al. 2012). Infants experiencing opioid withdrawal require resource‐intensive care with a higher average cost of $7800 and longer average length of stay (LOS) of 9 days, compared to $1100 and 2 days for newborns without opioid withdrawal (Centers for Disease Control and Prevention 2023). The precise number of infants experiencing withdrawal due to postnatal narcotic exposure remains unknown. However, infant withdrawal cases substantially affect neonatal healthcare utilization, prolong hospitalization, and increase economic burden. Therefore, improving the quality of care for these infants is critical to improving clinical outcomes and reducing health care costs (Gartley 2023).

## 2 Background

Infants experiencing substance withdrawal exhibit a variety of symptoms, including nervous system irritability, which usually requires hospitalization for symptom management (Hudak et al. 2012). Symptoms such as extreme irritability and inconsolable crying affect not only the infants but also the healthcare providers who care for them (Gartley 2023). Infants with NAS may exhibit a high‐pitched and nearly inconsolable cry that can contribute to nurses' frustration and burnout (Maguire et al. 2012; Murphy‐Oikonen et al. 2010). Neonatal intensive care unit (NICU) nurses often devote substantial portions of their shifts to consoling these infants while simultaneously managing other clinical responsibilities (Murphy‐Oikonen et al. 2010). Current approaches to nursing care for infants experiencing withdrawal, including caring for infants with NAS, emphasize addressing the clinical manifestations of withdrawal through symptom scoring, pharmacologic management, and nonpharmacologic soothing strategies, rather than supporting nurses in managing the emotional and workload burden associated with this care (Maguire et al. 2012). However, this emphasis can place NICU nurses in a more taxing position, as time‐consuming withdrawal care may compromise attention to other infant care needs (Maguire et al. 2012; Murphy‐Oikonen et al. 2010). Nurses have reported increased stress, feelings of being overwhelmed, and difficulties maintaining consistent care under such circumstances (Romisher et al. 2018). Consequently, the complex care demands of withdrawal may contribute missed nursing care (MNC), jeopardizing quality and safety of care.

Missed nursing care, defined as required nursing care that is omitted or delayed, often arises from excessive demands or insufficient resources (Kalisch et al. 2009). Missed nursing care has been consistently linked to adverse outcomes, including increased medication errors, patient readmission, and reduced patient satisfaction, underscoring its significance for patient safety and quality of care (Recio‐Saucedo et al. 2018). Over the past decade, research on MNC has expanded across diverse healthcare settings, including emergency departments (Amritzer et al. 2024), acute care settings (Assaye et al. 2022), intensive care units (Alanazi et al. 2023; Tubbs‐Cooley et al. 2025), post‐anesthesia care units (Kiekkas et al. 2021), nursing homes (Campagna et al. 2021), and rehabilitation settings (Hogh et al. 2018). In NICUs, MNC is influenced by both nurse‐ and shift‐level factors. At the individual nurse level, higher education and greater work experience were associated with lower rates of MNC (Kohanová et al. 2024), while personal attribute such as accountability have also been linked to reduced MNC (Drach‐Zahavy and Srulovici 2019). At the shift level, a higher nurse‐to‐infant ratio (Lake et al. 2017, 2018; Tubbs‐Cooley et al. 2019, 2025), elevated infant acuity (Lake et al. 2020; Tubbs‐Cooley et al. 2019), and increased subjective workload (Tubbs‐Cooley et al. 2019, 2025) contribute to an increased likelihood of MNC. Missed nursing care in NICUs has been associated with critical adverse infant outcomes, including poorer achievement of oral feeding, higher morbidity, and longer LOS (Lake et al. 2023; Tubbs‐Cooley et al. 2014). Despite extensive research on MNC across clinical settings and its various causes and consequences, little work has specifically examined MNC in infants experiencing substance withdrawal, a particularly vulnerable population. To address this gap, the present study investigates the relationship between infant withdrawal status and MNC in the NICU.

## 3 Aim

The study aim was to evaluate whether there was an association between shift‐level infant substance withdrawal status and infant‐specific MNC in NICUs.

*Hypothesis*: We hypothesized that infants identified as at risk for or actively experiencing substance withdrawal on a shift would be significantly more likely to experience MNC compared to infants not identified as at risk or actively withdrawing.

## 4 Methods

### 4.1 Design

This was a secondary analysis of data collected as part of a longitudinal, observational study designed to evaluate differential effects of objective and subjective nurse workload on shift‐level missed nursing care in NICUs using repeated nurse‐infant‐shift observations (Tubbs‐Cooley et al. 2025).

### 4.2 Study Setting and Sampling

The parent study was conducted in ten participating Level II, III, and IV NICUs across academic medical and pediatric hospital settings, representing a range of neonatal care acuity levels. Registered nurses were the primary participant group. Registered nurses in participating units who worked in direct patient care were recruited through convenience sampling. Participation was voluntary, and interested nurses self‐enrolled after responding to study recruitment materials. A multi‐method recruitment strategy was employed, and interested nurses reached out to the research team via phone, email, or by booking a virtual appointment through an online scheduling system. Infants constituted the second group of participants, and their clinical data were linked to participating nurses. Deidentified data were accessed via honest brokerage (Boyd et al. 2007), an institutional data management process that securely linked and de‐identified nurse and infant data before release to the study team, under a waiver of consent. Because missed nursing care items represent standard neonatal nursing practices and are not condition‐specific, demographic and clinical data from all infants with matched nurse data were included.

### 4.3 Inclusion and Exclusion Criteria

Registered nurse eligibility in the parent study included completion of unit orientation, provision of at least 16 h of direct patient care every 2 weeks, permanent employment in the participating NICU, and owning a smartphone. Nurses who held supervisory roles or had direct reports within the unit, although meeting the above criteria, were excluded. For infant participants, all infants with matched nurse data were eligible for inclusion unless caregivers declined permission for research use.

### 4.4 Measures

#### 4.4.1 Infant Substance Withdrawal

Infants who were identified as experiencing or being at risk for substance withdrawal were those either actively experiencing withdrawal symptoms or being at risk of withdrawal from opioids or other substances during a shift, based on nurse report. Nurses were asked to indicate whether the infant was actively withdrawing or at risk of withdrawal (yes/no) based on clinical observations during the shift when responding to a researcher‐designed survey administered at the end of each shift. No specific withdrawal assessment tool or standardized diagnostic criteria were provided within the survey question. Because withdrawal symptoms can fluctuate within and across shifts, nurse‐reported status was used to capture real‐time clinical presentation rather than relying solely on a static medical diagnosis. Similarly, withdrawal risk reflected nurses’ clinical assessment during the shift rather than predefined standardized criteria.

#### 4.4.2 Missed Nursing Care

Missed nursing care was assessed by a nurse report of 16 essential neonatal nursing care practices for individual assigned infants using a survey developed by the parent study researchers (Tubbs‐Cooley et al. 2025). These practices were selected based on prior empirical work and qualitative feedback from NICU nurses to ensure relevance to routine neonatal care. These assessments were collected at the end of every shift from enrolled nurses, with each survey being specific to an assigned infant. Each MNC item had five ordered response options: frequently missed, occasionally missed, rarely missed, never missed, and non‐applicable. The responses were dichotomized into “missed” or “not missed” categories for each item, consistent with the parent study's prior research and due to infrequent endorsement of the higher‐frequency missed care response categories, which limited variability across response levels for analysis (Tubbs‐Cooley et al. 2019, 2025). The responses “frequently missed,” “occasionally missed,” and “rarely missed” were classified as “missed,” whereas “not missed” was classified as “not missed.” When “non‐applicable” was indicated for a specific missed care item, the infant was removed from the analysis for that item. A global dichotomous “any missed care” indicator was constructed for each infant on each shift, signaling the occurrence of any of the 16 specified items reported as missed by the nurse during the shift. Consequently, we measured 17 types of MNC per infant per shift.

#### 4.4.3 Nurse Characteristics

Nurse characteristics assessed at study enrollment included sex, race, ethnicity, marital status, and professional qualifications included the highest degree in nursing, neonatal intensive care nursing certification, and years licensed as a registered nurse. Employment‐related characteristics, such as total weekly hours worked in direct patient care and working shift (day or night), were also assessed.

#### 4.4.4 Infant Characteristics

Infant characteristics included sex, race, ethnicity, gestational age, birth weight, and NICU length of stay. Shift‐level indicators included infant workload acuity scores derived from registered nurse documentation in the electronic health record (EHR) every 4 h, producing six measures within a 24‐h period (or three per 12‐h shift). We used the first acuity score recorded within each shift. Primary diagnosis data were extracted from EHR to determine whether the infant had an NAS diagnosis.

#### 4.4.5 Shift‐Level Workload Characteristics

Shift‐level workload characteristics included the maximum number of infants cared for during the shift, as an indicator of nurse‐to‐infant staffing ratio, and the occurrence of any critical incident involving the assigned infant or another infant during the shift was reported by enrolled nurses. Unit census at the start of the shift was also included as a shift‐level characteristic.

### 4.5 Data Collection

Nurse characteristics were collected at enrollment in the parent study. Research staff distributed survey links via text messages, and participants completed them in real time during enrollment meetings. Surveys recording infant withdrawal status, MNC, and other shift‐level nurse‐reported characteristics were collected at the end of each shift when data collection was active within a unit in the parent study. Using shift personnel rosters to ascertain a nurse participant's work schedule, the study team sent text messages to nurses approximately 1 h before the end of their shift to prompt survey completion. Nurses who did not submit data received two reminder text messages after the end of the shift and at 24 h post‐shift, and data were considered missing after 96 h. To reduce data collection burden, the standard end‐of‐shift text guided participants assigned more than two infants during a shift to report on the two infants occupying the highest numerically ordered bed spaces. This approach reduced the risk of selection bias by introducing a quasi‐randomization strategy to MNC reporting. Data quality procedures included routine review of survey completeness and verification of infant bed spaces, and follow‐up through the electronic messaging system occurred when needed.

Study initiation was staggered across the units between 2021 and 2022, and data collection for each unit occurred in four quarterly cycles each lasting 4 weeks (28 days), for a total of 16 weeks over 12 months. Participants completed surveys through MyCap, a mobile application linked to Research Electronic Data Capture (REDCap), using their personal smartphones. Each entry was time‐stamped and included unique identifiers for infants to create a link between nurse, infant, and shift. Data entered in MyCap were automatically transferred to REDCap, a secure, web‐based platform that supports validated data capture, audit trails, automated export to statistical software, and interoperability with external sources (Harris et al. 2009).

Infant clinical data were extracted from the EHR by an honest broker who matched it with nurse survey data and administrative data, replaced all identifiers with pseudo‐identifiers, and preserved the temporal ordering of data using pseudodates before releasing the dataset to investigators for analysis.

### 4.6 Data Analysis

Descriptive statistics, including frequencies, medians, and interquartiles were used to describe nurse and infant characteristics and MNC responses. Although data were collected longitudinally across repeated shifts, analyses focused on nurse‐infant‐shift observations because infant withdrawal status and associated care needs could vary across shifts. Given the cross‐classified multilevel data structure (infants and nurses nested within shifts within units and crossed between shifts), outcomes were modeled at the nurse‐infant‐shift level using logistic cross‐classified multilevel models (CCMMs) with random intercepts for units, nurses, and infants. A nurse‐infant‐shift was defined as the instance in which a given nurse provided care to a given infant during a given shift, allowing nurses and infants to appear in multiple shifts. Separate CCMMs were fit for each of the 16 MNC items and for the global MNC measure (Bhat 2000; Browne et al. 2001; Rasbash and Goldstein 1994; Raudenbush 1993). Analyses were conducted using STATA 18.0 and MLwiN (version 3.09). Consistent with the parent study analytic approach, iterative generalized least‐squares models ignoring cross‐classification were estimated first, and the fixed effects were used as start values for the CCMMs to reduce computation time (Leckie 2012). Final models were estimated using Bayesian methods implemented via Markov chain Monte Carlo procedures using the Metropolis–Hastings algorithm (Brown 2004).

To address the risk of inflated type I error due to multiple testing, *p*‐values were adjusted using the Benjamini–Hochberg procedure (Benjamini and Yekutieli 2001). The adjusted significance threshold, based on two‐tailed tests, was *p* < 0.005.

We controlled for nurse characteristics, including work experience, highest educational degree, NICU certification status, and work‐related situational factors, such as shift type (day/night), infant acuity score, nurse‐to‐infant staffing ratio, shift census, and the occurrence of any critical incident (e.g., code event) involving the assigned infant or another infant during the shift. In addition, NAS diagnosis status was included as an infant‐level covariate. To address the broader contextual conditions of the study period, which coincided with the COVID‐19 pandemic, we controlled for community‐level COVID‐19 severity, defined according to the Centers for Disease Control and Prevention (CDC) indicators of community transmission (Christie et al. 2021; Ohio Department of Health COVID‐19 Data Team 2023). Missing data were handled using listwise deletion, with the proportion of missing data ranging from 11.7% to 16.1% depending on the model.

## 5 Ethical Considerations

Written informed consent was obtained from all nurse participants at enrollment in the parent study, including permission to re‐analyze de‐identified data for new research purposes. A waiver of informed consent was approved for the use of de‐identified infant data, which were accessed through an honest broker. Infant records were excluded if caregivers declined permission for research use. The study was reviewed and approved by the Institutional Review Board of The Ohio State University (No. 2020H0054).

## 6 Results

### 6.1 General Characteristics of Participants

A total of 264 nurses enrolled in the parent study, while 249 nurses across the 10 NICUs contributed eligible shift‐level data and were included in the analytic sample for this study. Table 1 shows the characteristics of the nurses. Most nurses were female (95.2%, *n* = 237), and 89.2% (*n* = 222) identified as White. Most held a bachelor's degree or higher (91.2%, *n* = 227), and 22.5% (*n* = 56) possessed specialty nurse certification. In terms of the nurses’ working characteristics, 51.8% (*n* = 129) worked day shift, while 48.2% (*n* = 120) worked another shift. The average total NICU experience was 7.7 years (SD = 9.02).

**Table 1:** Nurse participant characteristics (*N* = 249).

	Number	%	Median	IQR
Female	237	95.18		
White Race	222	89.16		
Non‐Hispanic	239	95.98		
Bachelor's degree or higher	227	91.16		
Certified as a Neonatal Intensive Care Nurse				
No	193	77.51		
Yes	56	22.49		
Primary work schedule				
Day shift	129	51.81		
Night shift	104	41.77		
Others	16	6.43		
Years worked in NICU			5	2–11

Abbreviations: IQR, interquartile range; NICU, neonatal intensive care unit.

A total of 1477 infants were cared for by nurses in the final dataset, resulting in 11,807 nurse‐infant‐shifts. Of these, 894 shifts involved 179 unique infants who were reported by nurses to have withdrawal symptoms and/or to be at high risk of withdrawal. The remaining 10,913 nurse‐infant‐shifts involved 1,298 infants who were not reported as experiencing withdrawal symptoms and/or at risk.

Infant characteristics are summarized in Table 2. Among the 179 infants identified by nurses as experiencing or at high risk for withdrawal during at least one shift, 58.1% (*n* = 104) were male, and most infants in this group were White (69.3%, *n* = 113). The median gestational age was 255.5 days (IQR: 223–273), the median birth weight was 2599.9 grams (IQR: 1610.0–3041.9), and the median NICU length of stay was 23 days (IQR: 14–82). Of these 179 infants, 54 (30.2%) had NAS as their primary diagnosis.

**Table 2:** Infant characteristics (*N* = 1477).

	Infants experiencing or at risk of experiencing withdrawal in at least one shift (*n* = 179)				Infants *not* experiencing withdrawal in any shift (*n* = 1298)			
	Number	%	Median	IQR	Number	%	Median	IQR
Sex								
Male	104	58.10			690	53.20		
Female	75	41.90			607	46.80		
Race								
White	113	69.33			755	64.20		
Black	32	19.63			298	25.34		
Multi‐racial	15	9.20			74	6.29		
Others	3	1.84			49	4.17		
Ethnicity								
Non‐Hispanic	161	99.38			1,161	97.69		
Hispanic	1	0.62			19	1.60		
Others	0				10	0.84		
Gestational Age (day)			255.5	223–273			238	213–260
Birth Weight (gram)			2599.93	1609.97–3041.90			2050.95	1310.03–2889.95
NICU length of stay (day)			23	14–82			24	8–65
NAS as primary diagnosis	54	30.17			0			

Abbreviations: IQR, interquartile range; NICU, neonatal intensive care unit; NAS, neonatal abstinence syndrome.

Among the 1298 infants not identified by nurses as experiencing or at high risk for withdrawal during any shift, 53.2% (*n* = 690) were male. Most infants were White (64.2%, *n* = 755), followed by Black (25.3%, *n* = 298), and nearly all were non‐Hispanic (97.7%, *n* = 1161). The median gestational age was 238 days (IQR: 213–260), the median birth weight was 2051.0 grams (IQR: 1310.0–2890.0), and the median NICU length of stay was 24 days (IQR: 8–65).

### 6.2 Missed Nursing Care Descriptive Findings

Table 3 shows the distribution of 16 MNC items and global MNC by infant's withdrawal status during the shift. The number of applicable infant shifts for each missed nursing care is presented in Supplementary Table S1. For shifts involving infants experiencing withdrawal symptoms, the proportions of all missed care were higher across all 16 MNC items compared to shifts involving infants without withdrawal symptoms. The most frequently missed care items during shifts for infants experiencing withdrawal were lactation support to mother (35.3%), intravenous (IV) site assessment (33.9%), developmental care (33.6%), hourly surveillance (32.7%), and adherence to the ventilator‐associated pneumonia (VAP) care bundle (30.7%). In contrast, the most frequently missed care items during shifts with infants not experiencing withdrawal were IV site assessment (26.9%), hourly surveillance (24.0%), high‐risk drug assessment (23.3%), developmental care (20.1%), and lactation support to mothers (19.7%).

**Table 3:** Missed nursing care by infants’ withdrawal status during the shift when item was applicable to an infant's care.

	Infant Shifts Experiencing or At Risk of Experiencing Withdrawal (*n* = 894)		Infant Shifts *Not* Experiencing Withdrawal (*n* = 10,913)	
	*n*	%	*n*	%
Any missed care	507	56.71	4775	43.76
Missed lactation support to mother	24	35.29	699	19.72
Missed IV site assessment	105	33.87	869	26.89
Missed developmental care	295	33.56	2154	20.05
Missed hourly surveillance	292	32.74	2594	24.02
Missed VAP care bundle adherence	69	30.67	233	14.90
Missed airway tube position assessment	50	28.41	175	13.19
Missed high‐risk drug assessment	105	28.30	252	23.29
Missed bedside teaching with parents	91	28.17	1067	18.46
Missed oxygen titrated per protocol	91	25.00	805	16.01
Missed communication during handoffs	195	22.13	1485	13.76
Missed pain assessment per protocol	168	19.29	815	8.01
Missed oral feeding when baby cueing	98	18.77	531	8.75
Missed central line adherence	37	17.70	176	9.60
Missed parent invitation to baby's care	57	16.01	644	10.50
Missed infection prevention adherence	110	12.88	918	8.82
Missed breastmilk administration per protocol	2	1.14	121	1.79

*Note:* Infant shift indicates one unit of care per individual infant under a nurse's care during a single shift, among multiple infants assigned to that nurse. Percentages for each missed nursing care item were calculated based on infant shifts for which the item was applicable to the infant's care; therefore, denominators varied across items due to responses coded as “not applicable” (see Supplementary Table S1 for applicable infant shifts by item).

Abbreviations: IV, intravenous; VAP, ventilator‐associated pneumonia.

### 6.3 Results of Logistic Cross‐Classified Multilevel Analyses

Table 4 shows the results for the logistic cross‐classified multilevel analyses of infant's withdrawal status and the occurrence of the MNC. A forest plot displaying the adjusted odds ratios and 95% confidence intervals is provided in Supplementary Figure S1. Infant withdrawal status during the shift was associated with the occurrence of 7 MNCs items and global missed care measure. Specifically, infants experiencing withdrawal were 2.3 times more likely to experience missed pain assessment per protocol (OR = 2.29, *p* < 0.001, CI 1.69–3.06), 2.3 times more likely to experience missed airway tube position assessment (OR = 2.26, *p* = 0.002, CI 1.33–3.73), 1.8 times more likely to experience missed developmental care (OR = 1.81, *p* < 0.001, CI 1.44–2.24), and 1.8 times more likely to experience missed high‐risk drug assessment (e.g., narcotics, paralytics, and sedatives) (OR = 1.78, *p* = 0.002, CI 1.15–2.61), compared to infants not experiencing withdrawal. Additionally, they were 1.7 times more likely to experience missed oxygen titration per protocol (OR = 1.68, *p* = 0.004, CI 1.18–2.33), 1.4 times more likely to experience missed hourly surveillance (OR = 1.43, *p* = 0.002, CI 1.13–1.80), 1.5 times more likely to experience missed communication during handoffs (OR = 1.46, *p* = 0.003, CI 1.20–1.86), and 1.6 times more likely to experience any missed care (OR = 1.57, *p* < 0.001, 1.26–1.97), compared to infants not experiencing withdrawal. Withdrawal status was not significantly associated with other aspects of missed care, such as IV site assessment, feeding‐related practices (e.g., breastmilk administration per protocol, oral feeding when the infant was cueing), infection prevention, or parent‐related support activities (e.g., lactation support to mother, bedside teaching with parents, parent invitation to participate in the infant's care).

**Table 4:** Estimated association between infant withdrawal status and missed nursing cares.

Outcome variable	OR	SE	*p*	95% CI
Any missed care	1.57	0.18	< 0.001*	1.26, 1.97
Missed lactation support to mother	2.02	0.79	0.050	0.90, 3.95
Missed IV site assessment	1.33	0.24	0.073	0.91, 1.85
Missed developmental care	1.81	0.21	< 0.001*	1.44, 2.24
Missed hourly surveillance	1.43	0.17	0.002*	1.13, 1.80
Missed VAP care bundle adherence	1.34	0.33	0.136	0.80, 2.12
Missed airway tube position assessment	2.26	0.59	0.002*	1.33, 3.73
Missed high‐risk drug assessment	1.78	0.39	0.002*	1.15, 2.61
Missed bedside teaching with parents	1.70	0.31	0.006	1.17, 2.36
Missed oxygen titrated per protocol	1.68	0.30	0.004*	1.18, 2.33
Missed communication during handoffs	1.46	0.19	0.003*	1.20, 1.86
Missed pain assessment per protocol	2.29	0.35	< 0.001*	1.69, 3.06
Missed oral feeding when baby cueing	1.74	0.39	0.011	1.08, 2.59
Missed central line adherence	1.97	0.55	0.013	1.07, 3.26
Missed parent invitation to baby's care	0.99	0.21	0.444	0.63, 1.47
Missed infection prevention adherence	1.33	0.22	0.045	0.95, 1.78
Missed breastmilk administration per protocol	0.49	0.38	0.100	0.05, 1.45

*Note:* The reference category of the exposure variable was infant's status without withdrawal. Each model was controlled for nurse working years, nurse degree, neonatal intensive care nursing certification status, working shift, infant acuity, staffing ratio, starting census, covid severity, critical incident to this baby, critical incident to another baby, and whether neonatal abstinence syndrome was the infant's primary diagnosis.

Abbreviations: IV, intravenous; OR, odds ratio; SE, standard error; VAP, ventilator‐associated pneumonia; 95% CI, 95% confidence interval.

* *p* < 0.005, corrected for false discovery rate.

## 7 Discussion

We examined the relationship between infants’ withdrawal status and the occurrence of MNC in neonatal intensive care units. While MNC has been widely studied across diverse care settings, the potential vulnerability of infants experiencing withdrawal to MNC has received little attention. In this study, withdrawal status was associated with a higher likelihood of missed care in eight specific areas compared with infants not experiencing withdrawal: pain assessment, airway tube position assessment, developmental care, high‐risk drug assessment, oxygen titration, hourly surveillance, communication during handoffs, as well as in the global missed care measure.

Withdrawal status of infants was significantly associated with an increased likelihood of missed hourly surveillance compared to infants without withdrawal. Nurse surveillance is a deliberate and continuous process of collecting, interpreting, and synthesizing patient data to anticipate problems and guide decision‐making in patient care (Halverson and Scott Tilley 2022). Prior MNC studies have extensively examined focused or repetitive patient assessments, which can be considered closely aligned with the concept of surveillance. Prior NICU studies conducted across East Asian and European settings have reported substantial levels of missed nursing care related to assessment and monitoring, although findings have not been entirely consistent across studies (Kim and Chae 2022; Kohanová et al. 2024). Similar concerns regarding missed monitoring activities and delayed responses to patient care needs have also been reported in Middle Eastern NICU settings, suggesting that workload‐related missed care may represent a broader international challenge in neonatal nursing practice (Bakırlıoğlu et al. 2025). Infants experiencing withdrawal often require increased attention to monitor their fluctuating symptoms and to provide interventions that help relieve those symptoms (Maguire et al. 2012; Murphy‐Oikonen et al. 2010). Continuous surveillance is therefore crucial for ensuring quality of care for this vulnerable population. Although our data did not allow us to examine the causes for increased missed hourly surveillance care, in previous studies, researchers have cited urgent patient situations, clinical deterioration, increased workload and patient acuity, staffing limitation, and workflow‐related challenges as common contributors to MNC (Bakırlıoğlu et al. 2025; Kim and Chae 2022; Kohanová et al. 2024). While we could not determine causality, factors associated with nursing practice associated with nursing practice, work environment, staffing adequacy, and organizational support may be important to consider in future research, particularly in the context of sustaining high‐quality neonatal nursing care under conditions of high workload and care complexity (Batran et al. 2025).

We found that infants experiencing or at risk for experiencing withdrawal had a higher likelihood of missed pain assessment compared to those not experiencing or at risk of experiencing withdrawal. This is concerning, as these infants are likely to experience pain due to cessation of substance exposure, and opioid exposure may further increase the risk of altered brain maturation and pain perception (Maguire 2014; Oji‐Mmuo et al. 2020; Roychaudhuri et al. 2024). Routine pain assessment is required for all NICU infants, and effective pain assessment, prevention, and treatment have been recommended as the standard of care in NICU (American Academy of Pediatrics, Committee on Fetus and Newborn and Section on Surgery, Section on Anesthesiology and Pain Medicine, Canadian Pediatric Society, & Fetus and Newborn Committee 2006). Roychaudhuri et al. (2024) noted that existing pain assessment methods are insufficient for identifying infants’ pain and distress across gestational ages, even though several tools have been developed to assess withdrawal symptoms, such as the Finnegan Neonatal Abstinence Scoring System for infants with NAS and the NICU Network Neurobehavioral Scale (Finnegan et al. 1975; Lester and Tronick 2004). Despite this emphasis on pain and other related symptoms in the care of infants experiencing withdrawal (Chin Foo et al. 2021), our results indicate that pain assessment was more likely to be missed in these infants than in others. This finding holds significant implications, as these infants represent a vulnerable group with heightened susceptibility to pain (Roychaudhuri et al. 2024). In previous studies, researchers have highlighted barriers faced by nurses in effectively managing pain in NICU, including a lack of knowledge, fear, time constraints, and lack of confidence in utilizing in the pain assessment tools (Cong et al. 2013). Further investigation is necessary to comprehensively understand and address these barriers in the context of infants experiencing withdrawal.

In the present study, missed developmental care occurred more often for infants experiencing or at risk of experiencing withdrawal compared to those not experiencing or at risk of experiencing withdrawal. Developmental care encompasses a range of activities aimed at managing the environment and tailoring care to premature infants based on behavior observations, with the goal of promoting as much stability, organization, and competency in the infant as possible (Byers 2003). Researchers have explored developmental practices as a significant area of missed care in the NICU compared to other activities (Kim and Chae 2022). Yet, developmental care is widely recognized as one of the most crucial aspects of nursing care in the NICU, encompassing practices such as positive and timely sensory exposures, cyclic lighting, skin‐to‐skin contact, and optimal body positioning (Griffiths et al. 2019). Recent NICU literature has also highlighted the importance of nursing knowledge and supportive institutional practices in facilitating developmental care interventions, such as kangaroo mother care, in neonatal settings (Ayed et al. 2025). Prior research has shown that developmental interventions improve physiological and psychological outcomes, including weight gain and reduced length of stay (Haslbeck et al. 2020; Kumar 2024; Soleimani et al. 2020). For infants experiencing or at risk of experiencing withdrawal, developmental care often emphasizes minimizing overstimulation while providing appropriate forms of developmental support such as swaddling and skin‐to‐skin care (Mascarenhas et al. 2024). In future research, it will be important to explore the barriers nurses face in providing developmental care to infants experiencing or at risk of experiencing withdrawal and to strengthen education and resources that support the consistent delivery of tailored and effective practices for this population.

Infants experiencing withdrawal often receive high‐risk medications or undergo medication tapering, making consistent assessment of these drugs particularly important (Hudak et al. 2012). Prior results from NICU studies have shown that high‐risk medication verification is typically one of the least frequently missed care activities among general patient groups, suggesting that nurses usually prioritize this task (Kim and Chae 2022). In contrast, our findings indicate that high‐risk drug assessment represented one of the more frequently missed care activities during shifts involving infants experiencing or at risk of experiencing withdrawal, with these infants demonstrating a higher likelihood of experiencing missed assessments compared to infants without withdrawal. Although we could not examine underlying mechanisms, the clinical complexity and fluctuating symptoms often observed in withdrawal care may contribute to competing care demands that draw nurses’ attention toward immediate needs (Maguire et al. 2012; Murphy‐Oikonen et al. 2010). In such situations, high‐risk drug assessments may be more likely to be overlooked despite their importance. These findings underscore the need for further research on workflow dynamics in withdrawal care and strategies to support consistent adherence to high‐risk medication safety protocols.

Future studies should examine whether the presence of an infant with withdrawal in a nurse's assignment increases the likelihood of missed care for other assigned infants, potentially through workload redistribution or attentional drain. Infants with withdrawal symptoms often require prolonged soothing, repeated assessments, and frequent clinical decision‐making, all of which intensify nurses’ cognitive and emotional demands. Prior NICU research has also suggested that emotional and cognitive factors, including emotional intelligence, may support nurses’ clinical decision‐making and management of complex care demands in high‐intensity neonatal care environments (Ayed 2025b). In addition, nurses perceive substantially greater mental and physical workload when caring for this population (Maguire et al. 2012; Murphy‐Oikonen et al. 2010). Increased workload can reduce the time and attention available for completing required care activities, particularly those that are repetitive or time‐sensitive, such as hourly surveillance and protocol‐driven assessments (Kalisch and Williams 2009; Tubbs‐Cooley et al. 2025). Although we did not directly examine workload mechanisms, these established associations suggest that workload burden is likely an important contributor to missed care among infants experiencing or at risk of experiencing withdrawal. Researchers should evaluate how the unique demands of withdrawal care shape nurses’ workload, explore modifiable organizational factors, and test interventions that strengthen nurses’ capacity to deliver reliable care for this population of infants experiencing or at risk of experiencing withdrawal. Supportive approaches that promote nurses’ adaptive coping and sustained delivery of high‐quality care in high‐intensity NICU environments also warrant further investigation (Ayed 2025a).

## 8 Strengths and Limitations

In the parent study, data were integrated from various sources, including nurse surveys, infants’ EHRs, and institutional administrative data, down to the shift level. This comprehensive approach facilitated a detailed understanding of essential variables, linking nurse responses, individual infant‐shift level characteristics, and administrative information for each shift. Notably, this analysis is the first to examine the relationship between infants’ withdrawal status and MNC. The findings offer crucial insights into how an infant's withdrawal status affects the essential nursing care, thereby highlighting opportunities for quality improvement in the care of infants experiencing or at risk of experiencing withdrawal in the NICUs.

However, the study is not without limitations. This analysis is constrained by the available data, limiting our ability to examine underlying mechanisms or identify factors that may precede missed care. The reliance on nurse self‐reported data from a convenience sample and potential social desirability bias due to the sensitive nature of the study topic regarding missed nursing care could be considered as further limitations. Although the missed nursing care indicators used in this study were adapted from previously established measures (Kalisch and Williams 2009) and prior NICU research (Tubbs‐Cooley et al. 2025), future studies may continue to evaluate how these measures perform across diverse NICU settings and patient populations. Additionally, the dichotomization approach classified “rarely missed” responses as missed care, which may have captured relatively minor omissions alongside more clinically significant missed care events. Therefore, findings should be interpreted with consideration of the broad operationalization of missed nursing care used in this study. Future research using larger and more diverse samples, as well as study designs that better examine underlying mechanisms and causal pathways related to missed nursing care, may provide a more comprehensive understanding of the phenomenon under investigation.

## 9 Conclusion

We found that infants experiencing or at risk of experiencing substance withdrawal on a shift were more likely to experience missed nursing care compared to other infants in NICUs. Future researchers should specifically examine barriers to providing essential care to these infants. Nurse leaders should consider these results in the context of staffing and nursing resource allocation and consider organizational and system‐level strategies, such as enhancing unit teamwork and supporting parent engagement in care. Additionally, strategies may include targeted education on withdrawal care, integration of withdrawal risk into acuity‐based staffing approaches, and peer support initiatives to promote consistent care delivery for infants experiencing or at risk of experiencing withdrawal.

## Author Contributions

Sooyoung Kim conceptualized and designed the study, conducted the data analysis, interpreted the findings, and drafted the manuscript. Heather L. Tubbs‐Cooley contributed to study conception and design, provided overall scientific guidance, and critically reviewed and revised the manuscript. Adam C. Carle provided statistical mentorship and guidance on the analytic approach and interpretation of results. Rita Pickler and Jin Jun contributed to the development of the study and provided substantive feedback on the interpretation of findings and manuscript revisions. All authors reviewed and approved the final manuscript.

## Conflicts of Interest

The authors declare no conflicts of interest.

## Supporting information

Supporting File

## Data Availability

The data that support the findings of this study are available from the corresponding author upon reasonable request.
