# Supplementary material for: Missed Nursing Care in Neonatal Intensive Care Units for Infants Experiencing or at Risk of Experiencing Substance Withdrawal
Source: Res Nurs Health. 2026 Jul 3;49(5):668–78. doi: 10.1002/nur.70096 (PMC13383121; doi:10.1002/nur.70096)
Supplement: Supplementary file 1 [file NUR-49-668-s001.docx]

Supplementary Table S1. *Applicable Infant Shifts for Missed Nursing Care Items*

|  | Number of Applicable Infant Shifts |
| --- | --- |
| Any Missed Care | 5,282 |
| Missed Lactation Support to Mother | 723 |
| Missed IV Site Assessment | 974 |
| Missed Developmental Care | 2,449 |
| Missed Hourly Surveillance | 2,886 |
| Missed VAP Care Bundle Adherence | 302 |
| Missed Airway Tube Position Assessment | 225 |
| Missed High-risk Drug Assessment | 357 |
| Missed Bedside Teaching with Parents | 1,158 |
| Missed Oxygen Titrated per Protocol | 896 |
| Missed Communication during Handoffs | 1,680 |
| Missed Pain Assessment per Protocol | 983 |
| Missed Oral Feeding when Baby Cueing | 629 |
| Missed Central Line Adherence | 213 |
| Missed Parent Invitation to Baby’s Care | 701 |
| Missed Infection Prevention Adherence | 1,028 |
| Missed Breastmilk Administration per Protocol | 123 |

*Note.* IV, Intravenous; VAP, Ventilated Associated Pneumonia. Applicable infant shifts represent infant shifts for which each missed nursing care item was relevant to the infant’s care. Infant shifts in which care was eligible but not missed were not included in these 3counts.

Supplementary Figure S1. *Forest Plot of Adjusted Odds Ratios for Associations Between Infant Withdrawal Status and Missed Nursing Care Outcomes*

*Note*. IV, Intravenous; VAP, Ventilated Associated Pneumonia; OR = odds ratio. Circles represent adjusted ORs and horizontal lines represent 95% confidence intervals. The vertical dashed line indicates an OR of 1. Filled circles indicate statistical significance after false discovery rate correction (*p* < .005), whereas hollow circles indicate nonsignificant associations.
